# Targeting Cancer Gene Therapy with Magnetic Nanoparticles

**DOI:** 10.18632/oncotarget.490

**Published:** 2012-05-02

**Authors:** Charles Li, Linda Li, Andrew C. Keates

**Affiliations:** ^1^ Department of Medicine, Beth Israel Deaconess Medical Center, Harvard Medical School, Boston, MA 02215

**Keywords:** magnetic targeting, magnetic nanoparticles, xenograft tumors models

## Abstract

Recent advances in cancer genomics have opened up unlimited potential for treating cancer by directly targeting culprit genes. However, novel delivery methods are needed in order for this potential to be translated into clinically viable treatments for patients. Magnetic nanoparticle technology offers the potential to achieve selective and efficient delivery of therapeutic genes by using external magnetic fields, and also allows simultaneous imaging to monitor the delivery *in vivo*. Compared to conventional gene delivery strategies, this technique has been shown to significantly increase gene delivery to human xenograft tumors models, as well as various internal organs (e.g. liver, kidney) and the central nervous system. Magnetic nanoparticle technology, therefore, has the potential to turn the challenge of gene therapy *in vivo* into a new frontier for cancer treatment.

## TARGETED DELIVERY OF CANCER GENE THERAPY

Cancer, at its foundation, can be attributed to one or more malfunctioning genes. Gene therapy offers the possibility to directly address the root cause of cancer through the upregulation or downregulation of target genes and, thus, offers a wide range of potential treatment strategies for this disease. The most significant challenge to effective gene therapy is delivery *in vivo* [1-3]. Due to the presence of nucleases in the bloodstream and immune system recognition of foreign nucleic acids, DNA and RNA typically have very short half-lives in circulation [4]. Approaches that work *in vitro* are, therefore, hindered by the inability of therapeutic genes to reach their intended targets *in vivo*. Certain delivery methods have been shown to increase the half-life of gene therapeutics, most notably the addition of polyethylene glycol (PEG) to create “stealth” delivery methods. PEG is a polymer which, when complexed with nucleic acids, prevents proteins and nucleic acids from being recognized by the host immune system [5]. PEGylation also increases the hydrodynamic size of nucleic acids thereby reducing their renal clearance. However, even with increases in circulation half-life, genes and their carriers still may cause significant side effects due to insufficient selectivity in achieving targeted delivery with current delivery technologies [6].

Research on targeted gene delivery to cancer tissues has focused mostly on conjugation of the therapeutic gene payload to antibodies, or other ligands [7]. However, these approaches have been limited by challenges in identifying antigens or receptors that are specific to tumors. In this review article, we will focus on targeted gene delivery using magnetic nanoparticles.

## MAGNETIC NANOPARTICLE TECHNOLOGY

Magnetic nanoparticles that are used for drug delivery purposes are usually crystals between 5-20nm in diameter. These crystals are typically iron-based, most commonly magnetite or maghemite [8]. Several methods for synthesizing these crystals have been developed, the most common being co-precipitation of Fe(III) and Fe(II) [9].

For gene and drug delivery applications, magnetic nanoparticles are usually complexed with a delivery platform in order to encapsulate the drug or gene, and promote cell uptake. Delivery technologies that have been used with magnetic nanoparticles include polymeric, viral, as well as non-viral platforms. Moreover, several methods have been used to form these complexes including hydrophobic interactions [10] and electrostatic interactions [11].

**Figure 1:**
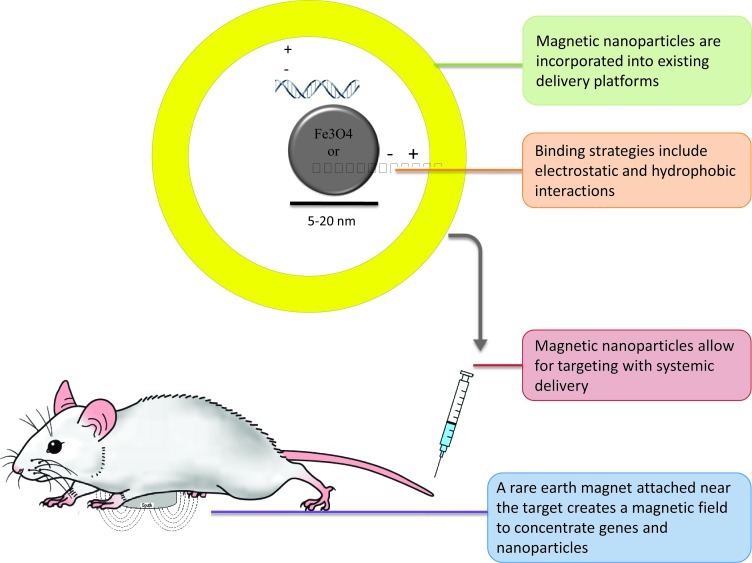
Overview of In Vivo Magnetically Targeted Gene Therapy

For *in vivo* targeting (Figure 1), treatment-nanoparticle complexes are injected intravenously, intraarterially or intraperitoneally, and an external magnet (usually a small rare earth magnet) is attached near the target region to create a localized magnetic field. As the drug circulates, the applied magnetic field acts on the magnetic nanoparticles (and attached drugs) to draw them into the surrounding tissue [12].

Compared to other delivery methods, magnetic nanoparticles have a number of advantages for drug delivery because of their demonstrated responsiveness to external magnetic fields, relative safety, and versatility. Magnetic nanoparticles have been approved for clinical use for over a decade as MRI contrast agents [13] and, therefore, are one of the better-understood nanotechnologies in terms of patient safety. In addition, since magnetic nanoparticles are compatible with a wide range of existing drug platforms, they can be used to effectively deliver a wide variety of therapeutic agents [14].

## TARGETED GENE DELIVERY *IN VIVO* USING MAGNETIC NANOPARTICLES

Magnetism-based targeted delivery was first described in 1978 [15]. However, methods similar to those used for drug delivery have significant potential to be used for gene therapy delivery. For these applications, though, the technology must be adapted to account for the size and charge of nucleic acids.

Of particular interest, magnetically targeted delivery offers a potential solution to the delivery issues currently hindering the development of effective gene therapies. For example, through the use of magnetic nanoparticles complexed with gene vectors, therapeutic genes can be selectively targeted by an external magnetic field to tumor sites in order to increase the concentration of therapeutic genes while decreasing the exposure in the rest of the body.

### Topical Delivery

Intratumoral injection, or injection near tumor sites, has been used to target tumors in clinical trials [16]. Magnetofection offers two potential advantages for topical delivery to tumors. First, it can increase the cellular uptake and retention of payloads at the injection site. Bhattarai et al delivered modified adenoviral vectors expressing LacZ conjugated with magnetic nanoparticles via direct injection into both the jejunum and the trachea [17]. Significantly higher levels of beta-galactosidase activity were found in the lung and jejunum in magnetic groups, indicating enhanced retention and expression under an external magnetic field. While this approach may not be applicable as a treatment modality for non-accessible tumors, it demonstrates the potential effectiveness of magnetofection for enhancing therapeutic gene retention with intratumoral injection.

A second advantage of magnetofection for topical delivery is tumor penetration. Current delivery methods cannot efficiently deliver therapeutic genes to all regions of tumors, especially the hypoxic center, due in part to the convoluted nature of the vasculature inside many tumors [18]. Moreover, this has been theorized to factor into the development of drug resistance. Topical delivery by magnetofection has been shown to increase both gene accumulation at the target tissue, as well as gene penetration into the smaller arteries in the tumor. Krotz et al targeted the cremaster muscle after femoral artery injection of fluorescently-labeled oligodesoxynucleotides and found increased fluorescence in the magnetic group, in addition to significant fluorescence in smaller arterioles [19]. The increased fluorescence in the smaller arterioles showed that magnetic targeting can increase penetration into the tissue, suggesting that this approach may enable increased penetration of tumor tissue via its blood supply.

#### Systemic Delivery

Systemic delivery is the ultimate goal of research on novel delivery technologies since it can be used broadly in various clinical indications and is convenient to administer. Furthermore, human tumor xenograft models in mice provide a straightforward way to test targeting *in vivo*, as well as externally-directed magnetofection. However, while human tumor xenografts can provide valuable insights into the effects of systemic delivery, these models are likely to significantly underestimate the complexity of targeted delivery in patients.

Thus far, the most promising application of magnetofection as an *in vivo* cancer therapy has been tested using a human tumor xenograft mouse model. Using a magnetic nanoparticle–lipid complex delivering a luciferase plasmid, Namiki et al found strong luciferase activity in animals treated with both nanoparticles and an external magnet, but no significant expression in other groups after delivering the same dose of genes [10]. This effect was confirmed in a second trial in tissue homogenates from tumors as evidenced by the presence of siRNA directed against the EGF receptor in the magnetized groups, and the lack of siRNA in the non-magnetized groups. Delivery of EGF receptor siRNA was associated with a 50% reduction in tumor mass compared to the control group, when targeted by an external magnet. This study also showed the differences in efficiency between different nanoplex formulations. When compared to a previously used magnetic complex, the newer formulation showed a 10-fold reduction in siRNA accumulation in the non-targeted organs, compared to the older formulation, suggesting improved selectivity in organ-targeting. This may have been due to the smaller size of the newer formulation. Taken together, these findings provide strong evidence of a clear therapeutic benefit in addition to a proof-of-concept for delivery of a reporter gene.

Monocytes have also been used as gene vectors for cancer therapy due to their natural affinity for tumors. In this approach, monocytes are first transfected *ex vivo* and then used to deliver therapeutic genes to tumors via the bloodstream. This method of gene delivery avoids the toxicity issues that arise for the use of non-endogenous delivery vehicles. However, previous attempts have been hindered by the challenge of targeting adequate numbers of cells to tumors [20]. A recent study by Muthana et al examined the ability of monocytes grown in the presence of magnetic nanoparticles to deliver genes to tumors [21]. The authors found 16.9±4.2% of tumor cells expressed GFP in the magnetized group, a significant increase over the 4.9±3.5% of tumor cells expressing GFP in the non-magnetized group. No data was shown on whether this led to a decrease in monocytes in the liver. While this study did not show any therapeutic benefit, since it delivered a marker gene, it demonstrated that magnetic nanoparticles can be used to improve the utility of cell-based gene vectors.

### DELIVERY TO INTERNAL ORGANS

While magnetic gene delivery works best with external organs or tissues, internal organs have been effectively targeted using external magnets [22]. Thus far, most studies investigating magnetic gene targeting of internal organs have used reporter genes. As Namiki et al has shown systems that have successfully delivered reporter genes can also be used to effectively deliver therapeutic genes once they have been optimized [10].

#### Liver

Gene therapy has shown promising results in treating hepatocellular carcinoma both *in vitro* and *in vivo*. These strategies include p53 gene replacement [23] and RNAi-mediated gene silencing [24]. In both trials, gene therapy only worked when genes were directly applied to the liver. Direct application allowed researchers to test the potential of gene therapy without a systemic delivery system. However, due to the invasiveness of intratumoral injection, a systemic delivery system may be more broadly applicable for clinical applications.

Magnetically targeted gene delivery has been shown to significantly improve systemic delivery efficiency to internal organs, and may provide a more viable method to delivery these promising gene therapies. Zheng et al placed an external magnet near the liver during transfection of a luciferase plasmid-magnetic liposome complex, and observed an increase in luciferase activity compared to a control group treated without an external magnet [11]. In other internal organs analyzed, luciferase activity was decreased when a magnet was placed over the liver, suggesting that magnetofection not only increases transfection at the target site, but also reduces exposure to other parts of the body.

#### Kidney

Kidneys have been targeted with magnetic gene therapy using similar conditions and techniques to the liver. Kumar et al secured an external magnet between the hind legs of mice to target chitosan-based magnetic nanoplexes capable of expressing eGFP to the kidneys and found far greater GFP fluorescence than a non-magnetized control [25]. However, this study did not address effects on tissue near the kidney, which may have also been affected by the magnet, or the long-term effects of magnetically enhanced delivery. Therefore, while this study showed that gene delivery to the kidney could be greatly enhanced by magnetic targeting, further studies on the effects on neighboring tissue will need to be performed.

### DELIVERY TO THE CENTRAL NERVOUS SYSTEM

Delivery to the central nervous system (CNS) faces the unique challenge posed by the blood brain barrier, an endothelial cell layer that prevents therapeutic genes and many other drugs from entering the CNS [26]. Two studies conducted on CNS-directed magnetic gene delivery have used direct injections to the CNS. Intracranial injection allows for gene therapy to reach the brain. However, a less invasive method for crossing the blood brain barrier would be preferable for clinical applications.

#### Spinal Cord

In the spinal cord, magnetic nanoparticle/PEI complexes have been shown to be targetable following intrathecal injection. However, circulation and diffusion of the cerebrospinal fluid can reduce transfection at the injection site [27]. Using a magnetic nanoplex to deliver a pCAG-luc plasmid, Song et al was not only able to increase transfection at the injection site in the lumbar region, but was also able to specifically target genes in the cervical region when the magnet was moved, as measured by increased luciferase activity [27]. For spinal tumors, this technique offers a unique method for targeting various regions of the spine by increasing the effect of a therapy at the tumor site and reducing exposure at other regions. Moreover, this approach potentially allows for the treatment of tumors in the cervical and thoracic regions of the spine by means of a lumbar puncture.

#### Brain

Thus far, no studies have been performed on magnetic gene delivery to the developed brain. However, adenoviral vectors expressing GFP have been conjugated to magnetic nanoparticles and successfully targeted to a particular hemisphere of the rat embryonic brain [28]. After direct injection into the 3^rd^ ventrical of a rat embryo *in utero*, Sapet et al selectively delivered GFP to one side of the embryonic brain after application of an external magnet, as detected by fluorescence microscopy [28]. While this study showed promise for targeted delivery inside the brain, further studies will be necessary to determine whether this can approach can be adapted for delivery of therapeutic genes to a fully developed adult brain.

### FUTURE PERSPECTIVES

Research on magnetically targeted gene therapy, while relatively new, has shown significant potential to unleash the promise of gene therapy. However, many challenges remain, the most significant of which is formulation. As Namiki et al showed changes in formulation can lead to large changes in selectivity and in gene expression [10].

The external guiding magnet is another aspect that may benefit from optimization. Current methods typically use off the shelf magnets, without optimization of magnetic field strength or placement. The targeting efficiency of this therapy is likely to be significantly improved by the optimization of these important parameters.

Once these issues are ironed out, the versatility inherent to the nanoplex platform holds the potential to combine multiple functions into a single treatment. Various research groups have recently used the MRI contrast properties of magnetic nanoparticles to monitor the biodistribution of these nanoplexes [12]. This ability allows for more efficient optimization of the vehicle by revealing where these particles are concentrated.

With the addition of targeting components as part of the nanoplex, therapy and diagnosis can be combined [29]. Targeting ligands such as folate take advantage of the higher rate of folate uptake by cancer cells to increase selectivity [30]. Moreover, antibodies such as Herceptin (which binds to HER2/neu receptor) can be used as well [31], allowing for even greater selectivity and, therefore, better potential diagnostics with MRI monitoring.

Most importantly, a well-formulated nanoplex may improve the use of current therapies. Magnetic targeting, as shown by these studies, has the ability to increase the efficiency of gene therapy, and may potentially allow for the use of promising therapies that are limited by the high dosages currently required.
